# Polymorphic Ga_2_S_3_ nanowires: phase-controlled growth and crystal structure calculations†

**DOI:** 10.1039/d2na00265e

**Published:** 2022-07-01

**Authors:** Kidong Park, Doyeon Kim, Tekalign Terfa Debela, Mourad Boujnah, Getasew Mulualem Zewdie, Jaemin Seo, Ik Seon Kwon, In Hye Kwak, Minkyung Jung, Jeunghee Park, Hong Seok Kang

**Affiliations:** Department of Advanced Materials Chemistry, Korea University Sejong 339-700 Republic of Korea parkjh@korea.ac.kr; Institute for Application of Advanced Materials, Jeonju University Chonbuk 55069 Republic of Korea; DGIST Research Institute, DGIST Daegu 42988 Republic of Korea; Department of Nano and Advanced Materials, Jeonju University Chonju Chonbuk 55069 Republic of Korea hsk@jj.ac.kr

## Abstract

The polymorphism of nanostructures is of paramount importance for many promising applications in high-performance nanodevices. We report the chemical vapor deposition synthesis of Ga_2_S_3_ nanowires (NWs) that show the consecutive phase transitions of monoclinic (M) → hexagonal (H) → wurtzite (W) → zinc blende (C) when lowering the growth temperature from 850 to 600 °C. At the highest temperature, single-crystalline NWs were grown in the thermodynamically stable M phase. Two types of H phase exhibited 1.8 nm periodic superlattice structures owing to the distinctively ordered Ga sites. They consisted of three rotational variants of the M phase along the growth direction ([001]_M_ = [0001]_H/W_) but with different sequences in the variants. The phases shared the same crystallographic axis within the NWs, producing novel core–shell structures to illustrate the phase evolution. The relative stabilities of these phases were predicted using density functional theory calculations, and the results support the successive phase evolution. Photodetector devices based on the p-type M and H phase Ga_2_S_3_ NWs showed excellent UV photoresponse performance.

## Abstract 2

Polymorphic Ga2S3 nanowires grown *via* chemical vapor deposition exhibit distinctive evolution of various crystal structures.

## Graphical abstract

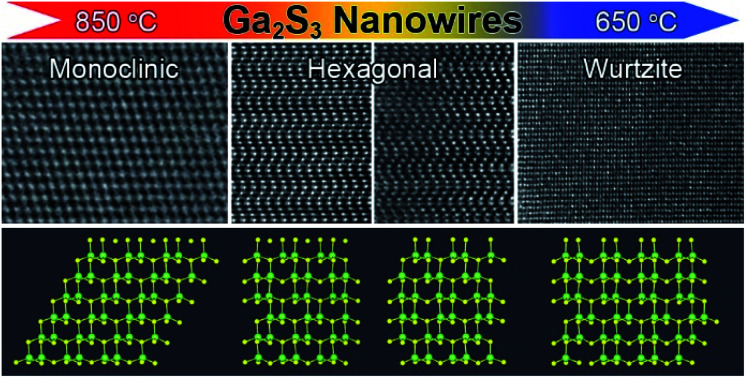

## 1. Introduction

Nanowires (NWs) have emerged as well-defined one-dimensional building blocks of next-generation nanodevices.^1,2^ Unlike the thermodynamically stable phase of their bulk counterparts, NWs often adopt metastable phases. Therefore, the controlled synthesis of metastable structures and their characterization are very attractive topics. Gallium sesquisulfide (Ga_2_S_3_) is a semiconductor with a wide bandgap (*E*_g_ = ∼3 eV at room temperature).^3^ It has a defective crystal structure, in which one-third of the Ga sites are vacant and the S atoms are arranged almost perfectly in a closely packed hexagonal lattice.^3–12^ The different ordering in the Ga sublattice results in a polymorphism of four crystalline phases: the monoclinic α′ phase (space group *Bb* or *Cc*), hexagonal α phase (*P*6_1_), wurtzite-type β phase (*P*6_3_*mc*), and zinc blende (also known as sphalerite)-type γ phase (F43m). The α′ phase is exactly stoichiometric and thermodynamically stable. Conversion of the α′ phase into other phases has been observed under a small number of S vacancies (<0.03%) and high-temperature modifications.^8,11,12^ Both the α and α′ phases are categorized by the superstructures of the β phase. The α and β phases exist at temperatures higher than that of the γ phase.

Previous studies of Ga_2_S_3_ bulk crystals or thin films (α′ phase) have focused on their optical properties, such as strong photoluminescence (PL) ranging from near-infrared to blue (400 nm) due to vacancy defects,^13–17^ excellent photoconductivity upon UV or blue irradiation,^15,16,18,19^ and infrared second-order nonlinear optical properties.^20^ The synthesis of α′-Ga_2_S_3_ NWs has been demonstrated using various methods, including chemical vapor deposition (CVD) and sulfurization of Ga_2_O_3_ NWs pre-synthesized by CVD or hydrothermal reaction.^21–25^ The Sutter group synthesized γ-Ga_2_S_3_ nanotubes by sulfurization of GaAs NWs.^26^ Since graphene-like two-dimensional (2D) layered materials have attracted much attention, the existence of 2D hexagonal crystal structures of Ga_2_S_3_ was theoretically predicted.^27,28^ Experimental studies demonstrated the synthesis of atomically thin layers of α′-, β-, and γ-Ga_2_S_3_ and their high-sensitivity UV photodetection.^29–31^ Nevertheless, there are few studies on the phase control of Ga_2_S_3_ nanostructures.

This work examines the phase-controlled growth of Ga_2_S_3_ NWs using CVD. Ga_2_S_3_ NWs with a monoclinic (α′) phase were successfully grown at 850 °C. As the growth temperature was gradually lowered to 600 °C, there was a sequential transition to the hexagonal (α) → wurtzite (β) → zinc blende (sphalerite) cubic (γ) phases. Atomically resolved transmission electron microscopy (TEM) revealed novel superlattice structures of the hexagonal phase in two different Ga arrangements, which were observed for the first time. The polymorphism produced distinctive core–shell structures in which the phases shared the same crystallographic axis. From this point on, the monoclinic, hexagonal, wurtzite, and zinc blende phases are respectively referred to by the English letters M, H, W, and C, instead of the Greek letters. First-principles calculations were performed on various polymorphic crystal structures involved in the phase evolution, and the results support the experimental results. Using the grown NWs, we fabricated photodetector devices and demonstrated their electrical and photoelectrical properties. Since polymorphism could be an important subject for optoelectronic devices, our work provides intriguing insights into their promising applications.

## 2. Experimental

### 2.1. Synthesis

Ga_2_S_3_ powders were placed in ceramic boats, which are loaded inside a quartz tube CVD reactor that is heated using an electrical furnace. A silicon (Si) substrate, on which a 5 nm-thick Au film was deposited, was positioned at a distance of 18 cm away from the powder source. The reactor was evacuated using a mechanical pump. Then argon gas is continuously supplied at a rate of 500 sccm during growth while the pressure maintains below 20 torr. The temperature of the powder sources is set to 900–950 °C. The substrate is maintained at 600–850 °C to synthesize the nanowire. Detailed experimental and methods are described in the ESI.†

### 2.2. Calculation

First-principles calculations were performed using density functional theory (DFT) as implemented in the Vienna ab-initio simulation package (VASP).^32,33^ Electron–ion interactions were described using the projector-augmented wave (PAW) method with a plane-wave kinetic energy cutoff of 520 eV.^34^ For the exchange–correlation functional, the generalized gradient approximation (GGA) suggested by Perdew, Burke, and Ernzerhof (PBE) was employed.^35^ Structure optimization (both ion and lattice relaxation) was performed until the average force was <0.01 eV Å^−1^ and the final energy change was <10^−8^ eV. Uniform *k*-point meshes with a reciprocal-space resolution of 2π × 0.32 Å^−1^ were used. To calculate the total density of states (DOS) and band gap (*E*_g_), modified Becke-Johnson (mBJ) exchange functional developed by Tran and Blaha, was adopted using the PBE correlation functional.^36^

## 3. Results and discussion

**Fig. 1:**
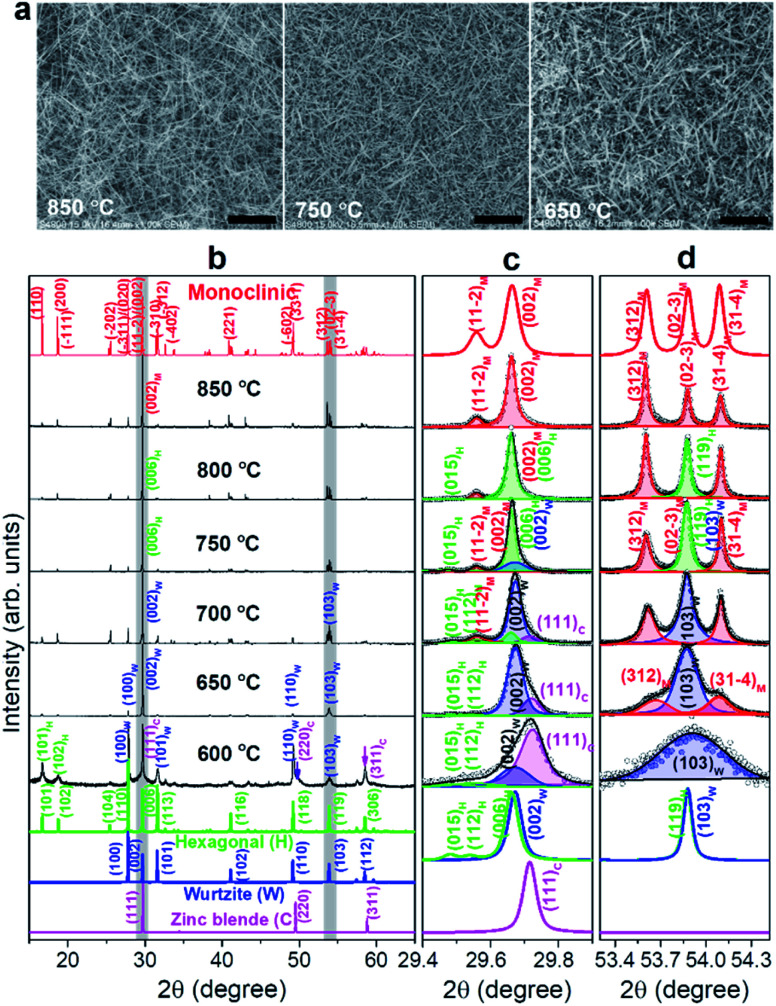
(a) SEM images of Ga_2_S_3_ NWs grown on a Si substrate at 850, 750, and 650 °C. The scale bars represent 2 μm. (b) XRD patterns of Ga_2_S_3_ NW samples synthesized at different temperatures (600–850 °C). The peaks were matched to the monoclinic (*a* = 11.130 Å, *b* = 6.406 Å, *c* = 7.037 Å, *β* = 121.22°), hexagonal (*a* = 6.412 Å and *c* = 18.050 Å), wurtzite (*a* = 3.702 Å and *c* = 6.017 Å), and zinc blende phases (*a* = 5.207 Å). The subscripts “M”, “H”, “W”, and “C” denote indices in the monoclinic (α′), hexagonal (α), wurtzite (β), and zinc blende cubic (γ) systems, respectively. Lattice parameters of the M, H, W, and C phases are close to the respective reference values for JCPDS No. 76-0752 [space group *Cc* (9), *a* = 11.14 Å, *b* = 6.411 Å, *c* = 7.038 Å, *β* = 121.22°], JCPDS No. 84-1440 [*P*6_1_ (169), *a* = 6.385 Å and *c* = 18.040 Å], JCPDS No. 74-0145 [*P*6_3_*mc*, *a* = 3.678 Å and *c* = 6.016 Å], and JCPDS No. 43-0916 [F43m, *a* = 5.215 Å]. Magnified peaks at (c) 2*θ* = 29.4°–29.9° and (d) 53.3°–54.4°. The experimental data (open circles) are fitted by a Voigt function, and the sum of the resolved bands (red: M phase, green: H, blue: W phase, magenta: C phase) is represented by the black line.

Fig. 1a shows the scanning electron microscopy (SEM) images of Ga_2_S_3_ NWs that were densely grown on a Si substrate at 850, 750, ad 650 °C. SEM images of all samples are shown in Fig. S1.† As the growth temperature decreased to 600 °C, the density of the NWs on the substrates decreased significantly. At 850 °C. the NWs had a straight and smooth surface. The high-resolution transmission electron microscopy (HRTEM) images are shown in Fig. S2.† The diameter of the NWs (average value: 150 nm) was uniform along a few tens of micrometers in length. As the temperature decreased to 650 °C, the NW morphology changed gradually to a tapered belt shape. The width (average value: 100 nm) was gradually reduced by more than half when approaching the tip. In Fig. S2,† we proposed a kinetically controlled growth model for the morphology change.

The X-ray diffraction (XRD) patterns of the grown Ga_2_S_3_ NWs are shown in Fig. 1b. The XRD peaks of the samples grown at 850 °C are matched to the M phase. As the temperature decreased, some of the peaks were diminished or shifted in position because of the incorporation of other polymorphic phases. At 650 °C, the XRD pattern became closer to that of the W phase. The XRD peaks at 600 °C indicated the C phase. Fig. S3† displays the correlation that links the crystallographic axes of the M, H, W, and C phase unit cells. Neglecting the small distortion of the M phase, the following relationships hold: 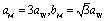, and 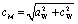. A correlation exists between the H and W phases, 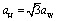 and *c*_H_ = 3*c*_W_. The M and H phases are superstructures of the W phase. Because the H phase can also be a superstructure of the M phase, its XRD includes the peaks of the M phase.

The peaks at 2*θ* = 29.4°–29.9° are magnified in Fig. 1c. The samples grown at 850 °C exhibited (002)_M_ and (112̄)_M_ peaks at 29.66° and 29.56°, respectively. As the growth temperature decreases to 800 °C, the (015)_H_ peak appeared at 29.47°, suggesting an incorporation of the H phase. The peak position of (006)_H_ appears to be coincident with that of (002)_W_. At 750 °C, the asymmetric band was resolved into two peaks corresponding to (002)_M_ (at 29.66°) and (006)_H_/(002)_W_ (at 29.67°). At both 700 and 650 °C, the main (29.67°) and shoulder (29.72°) peaks were assigned to the (002)_W_ and (111)_C_ peaks, respectively, indicating that the W phase became the major phase and the C phase started to emerge. At 600 °C, the main peak (29.72°) was assigned to (111)_C_ and the minor peak (29.67°) to (002)_W_. The (111)_C_ peak at the higher angle than the (001)_W_ indicates that the lattice constant of C phase is smaller than that of W phase by 0.5%, based on the W–C phase relationship. It means that the Ga–Ga distance is shorter than that of H and W phases.

Fig. 1d shows the (312)_M_, (023̄)_M_, and (314̄)_M_ peaks of samples grown at 850 °C. As the temperature decreased to 650 °C, the peaks became broader and the central (023̄)_M_ peak became more intense due to the inclusion of the (119)_H_/(103)_W_ peaks. At 600 °C, all peaks merged into the (103)_W_ peak. The intensity also decreased significantly, implying that the C phase had become the major phase. This XRD peak analysis provides definite evidence for the M → H → W → C phase evolution, consistent with previous studies on bulk materials.^8,11^

The XPS and Raman spectra shown in Fig. S4 and S5,† respectively, confirming that the electronic structures are almost same for these samples. UV-visible absorption and PL spectra provide the similar *E*_g_ value (3.0 eV) for all samples, which is consistent with previous works (Fig. S6†).^3,16,17,19^

**Fig. 2:**
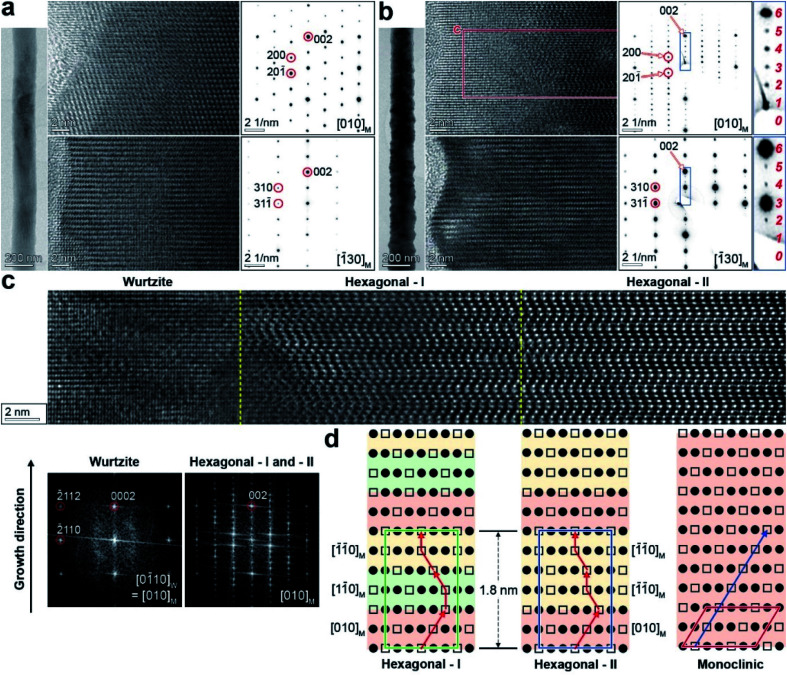
Lattice-resolved TEM images and the corresponding SAED patterns of Ga_2_S_3_ NW grown at (a) 850 °C and (b) 750 °C at the zone axes of [010]_M_ and [1̄30]_M_. The SAED patterns of (a) show the single-crystalline M phase. The rightmost column of (b) shows 1.8 nm-periodic superlattice structures with six divisions between the (002)_M_ spots. (c) Atomic-scale TEM images for the marked region in (b) with an extension to the core part (zone axis = [010]_M_). FFT images were generated for the wurtzite phase shell and the hexagonal phase middle/core parts. (d) Schematic diagrams of the atomic arrangement for the H-I (middle), H-II (core), and M phases. The filled circles and hollow squares represent sites occupied by Ga and vacancies, respectively. The arrow lines indicate the Ga vacancy sites. The unit cell of each phase is outlined in color. The unit cell of the H phase is comprised of six atomic layers along the [0001]_H_ (=[001]_M_) direction. Domains of the M phase variants (projected at the {010} zone axis) are distinguished from each other using different colors: [010]_M_ (red), [11̄0]_M_ (green), and 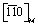 (yellow) for H-I; [010]_M_ (red), 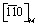 (yellow), and 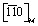 (yellow) for H-II.

Fig. 2a shows the HRTEM images (measured using high-voltage TEM at 1.2 MV) and SAED patterns of M phase Ga_2_S_3_ NW (grown at 850 °C) at two zone axes ([010]_M_ and [1̄30]_M_). The NWs usually had a straight and smooth surface. The diameter (170 nm) was uniform along the growth direction of [001]_M_. As the TEM grid holder was tilted 30° to rotate the NW, the zone axis could be altered from [010]_M_ to [1̄30]_M_. The same SAED pattern was generated for the entire NW, confirming its single-crystalline nature. We note the following typical relation in crystallographic axes of the four phases: [103]_M_ (normal to the shared (001)_M_ plane) = [0001̄]_H_ = [0001]_W_ = [111]_C_, [010]_M_ = [1̄21̄0]_H_ = [01̄10]_W_ = [112̄]_C_, and [1̄30]_M_ = [011̄0]_H_ = [12̄10]_W_ = [011̄]_C_.

Fig. 2b shows the data for the Ga_2_S_3_ NWs grown at 750 °C. These NWs typically have a rough surface. The SAED pattern was indexed using the M phase, in order to show that they were measured at the same zone axes as those in Fig. 2a. The NWs had a superlattice structure with a uniform period of 1.8 nm, corresponding to the H phase (*c* = 18.05 Å).^9^ The growth direction was identified as [001]_M_ = [0001]_H/W_. In both SAED patterns at the zone axes of [010]_M_ and [1̄30]_M_, the diffraction spots from two atomic layers along the [001]_M_ growth direction are divided into six parts (the magnified images on the right).

Fig. 2c shows the lattice-resolved image of the marked area (8 nm × 50 nm with an extension to the core) in Fig. 2b (zone axis = [010]_M_). The middle and core parts (right) show lattice fringes that are distinct from the shell (left). The bright atomic sites corresponding to Ga vacancies are ordered in a zigzag pattern. Fast Fourier transform (FFT) images were generated for each part (bottom), and they revealed a single-crystalline W phase in the shell. The FFT image of the middle and core parts is the same as the corresponding SAED pattern.

The atomic arrangement in the middle and core parts was inferred from the TEM images at the zone axis of [1̄21̄0]_H_ (equivalent to [010]_M_), as shown in Fig. 2d. One-third of the Ga sites on the (001)_M_ planes are vacant, as marked by the hollow squares, where the occupied Ga atoms are marked by the filled circles. The unit cell of the H phase is comprised of six atomic layers along the [0001]_H_ (=[001]_M_) direction. The ordered Ga vacancy sites along the growth direction produce a zigzag pattern, as indicated by the arrow line. They are distinctive from the one-directional Ga vacancies of the M phase, as shown in the right panel. The atomic arrangement in the middle part is the same as that of the H phase reported for bulk materials.^7,9,11^ This H phase is referred to as the “H-I” phase. The superlattice structure can originate from the periodic stacking of the M phase variants separated by 120° rotations. Fig. S7† shows the structural models of the M phase for the [010]_M_, [11̄0]_M_, and 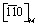 projections by virtue of rotating 120° around the [001]_M_ growth direction, which produces indistinguishable SAED and FFT images. The atomic arrangements of the three layers exactly match those of the [010]_M_, [11̄0]_M_, and 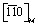 variants. The Ga vacancy sites are ordered by a sixfold screw rotation along the growth direction.

The superlattice structures at the core part have the same unit cell as that of the H phase, based on the SAED and FFT images. However, they have different atomic arrangements from those of the H phase shown in the middle part. This superlattice structure consisted of the three M phase variants ([010]_M_, 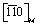 and 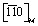) projected by 120° and 0° rotations around the [001]_M_ growth direction. Fig. S7† shows the structural models of the M phase projected at the [010]_M_ and 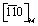 zone axes. The Ga vacancy sites don't show a screw type ordering. This new superlattice structure has never been reported before, and we call it the “H-II” phase. The rough surface of the NWs probably resulted from the stacking of rotated M-phase variants. Structural disorders originating from the random stacking of rotational variants were previously reported for the M phase of Li- or Na-intercalated transition metal oxide materials.^37,38^ However, to the best of our knowledge, uniform stacking of the variants has never been observed for M-phase materials.

**Fig. 3:**
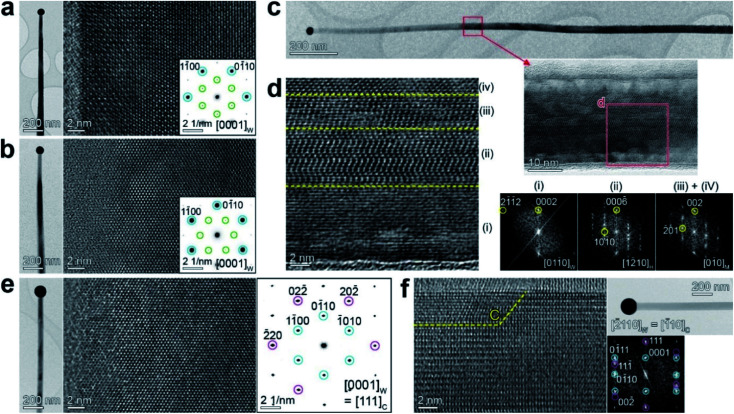
Lattice-resolved TEM images and the corresponding SAED (or FFT) patterns of tapered belt-like Ga_2_S_3_ NWs grown at 650 °C. (a), (b) and (e) The basal plane at the zone axis of [0001]_W/H_. The side plane at the zone axis of (c) and (d) [011̄0]_W_ = [12̄10]_H_ = [01̄0]_M_ and (f) [2̄110]_W_ = [1̄10]_C_. In the SAED patterns of (a) and (b), the {01̄10}_W_ (strong) and {011̄0}_H_ (weak) spots are marked by blue and green circles, respectively. The growth direction was identified as [12̄10]_W_ in (a) and [01̄10]_W_ in (b). The FFT images in (d) correspond to regions (i)–(iv) at the zone axis of (i) [011̄0]_W_, (ii) [12̄10]_H_, and (iii)/(iv) [01̄0]_M_. In the SAED pattern of (e), the {01̄10}_W_ and {22̄0}_C_ spots are marked by blue and magenta circles, respectively. In the FFT images of (f), the spots of W and C phases are also marked by blue and magenta circles, respectively.

Fig. 3a and b show the HRTEM image and the corresponding SAED pattern of Ga_2_S_3_ NWs grown at 650 °C, measured at the zone axis of [0001]_W_. The “W” index was used here since it is the major phase, which also agrees with the XRD data (with minor H and C phases). The growth directions were identified as [12̄10]_W_ and [01̄10]_W_, respectively. The electron beam used in TEM was projected onto the basal plane of the belt-like NW with a tapered morphology. The SAED patterns show the stronger W and weaker H phase spots, indicating that the two NWs have different growth directions, but both contain the H phase.

Fig. 3c shows the HRTEM and the corresponding FFT images measured at the zone axis of [011̄0]_W_. The electron beam was projected onto the side facet of the belt-like NWs and perpendicular to the basal plane. The magnified image (marked area) shows the superlattice-structured H phase at the center. Fig. 3d clearly reveals the superlattice structure of the H phase in region (ii). FFT images were generated for the regions labeled (i)–(iv). The shell part (i) and the inner part (ii) show the W and H phases, respectively, confirming the coexistence of these two phases. The atomic arrangement of the H phase corresponds to a mixture of H-I and H-II structures. The core parts (iii) and (iv) were assigned to the twinned M phase at the [01̄0]_M_ and [110]_M_ projections that overlapped after 120° rotations. The NW exhibits the W phase in the shell, the H phase in the middle, and the M phase in the core. The most stable M phase in the core and the metastable phase in the shell are supported by our growth model, in which the growth of shell parts is more driven under kinetically controlled conditions (see Fig. S3†).

The HRTEM image and SAED pattern were measured for another NW at the zone axis of [0001]_W_ (Fig. 3e). This NW has a growth direction of [01̄10]_W_. The W and C phase SAED spots indicate that the C phase coexists with the W phase. Fig. 3f shows the HRTEM and corresponding FFT images measured by projecting the electron beam onto the side plane of the belt-like NW. The lattice fringes of the C phase were found at the surface (the region marked with the letter “C”). The growth direction was identified as [01̄10]_W_ or [112̄]_C_. We monitored the growth direction for a few tens of NWs and found that 80% and 20% of them grew along [01̄10]_W_ and [12̄10]_W_, respectively.

Analysis results for the sample grown at 650 °C can be summarized as follows. (i) The tapered belt-like NWs show M → H → W → C phase evolution from the core to the shell, and the phases share the same crystallographic axis. The core–shell structures provide a panoramic view of the phase transition. (ii) The growth direction is [01̄10]_W_ or [12̄10]_W_, equivalent to [010]_M_ and [3̄31]_M_, respectively, unlike the [001]_M_ growth direction of the M-phase NWs grown at 850 °C (shown in Fig. 2a). Rao *et al.* reported the [100]_M_ growth direction for Ga_2_S_3_ NWs with a twinned structure synthesized using CVD at 650–800 °C.^21^ This growth direction is different from any of the directions in our sample, even if we convert the H (or W) phase axis into the M phase axis. Fig. S8† shows a schematic of the evolution of the growth direction, with a plausible model to explain the result.

**Fig. 4:**
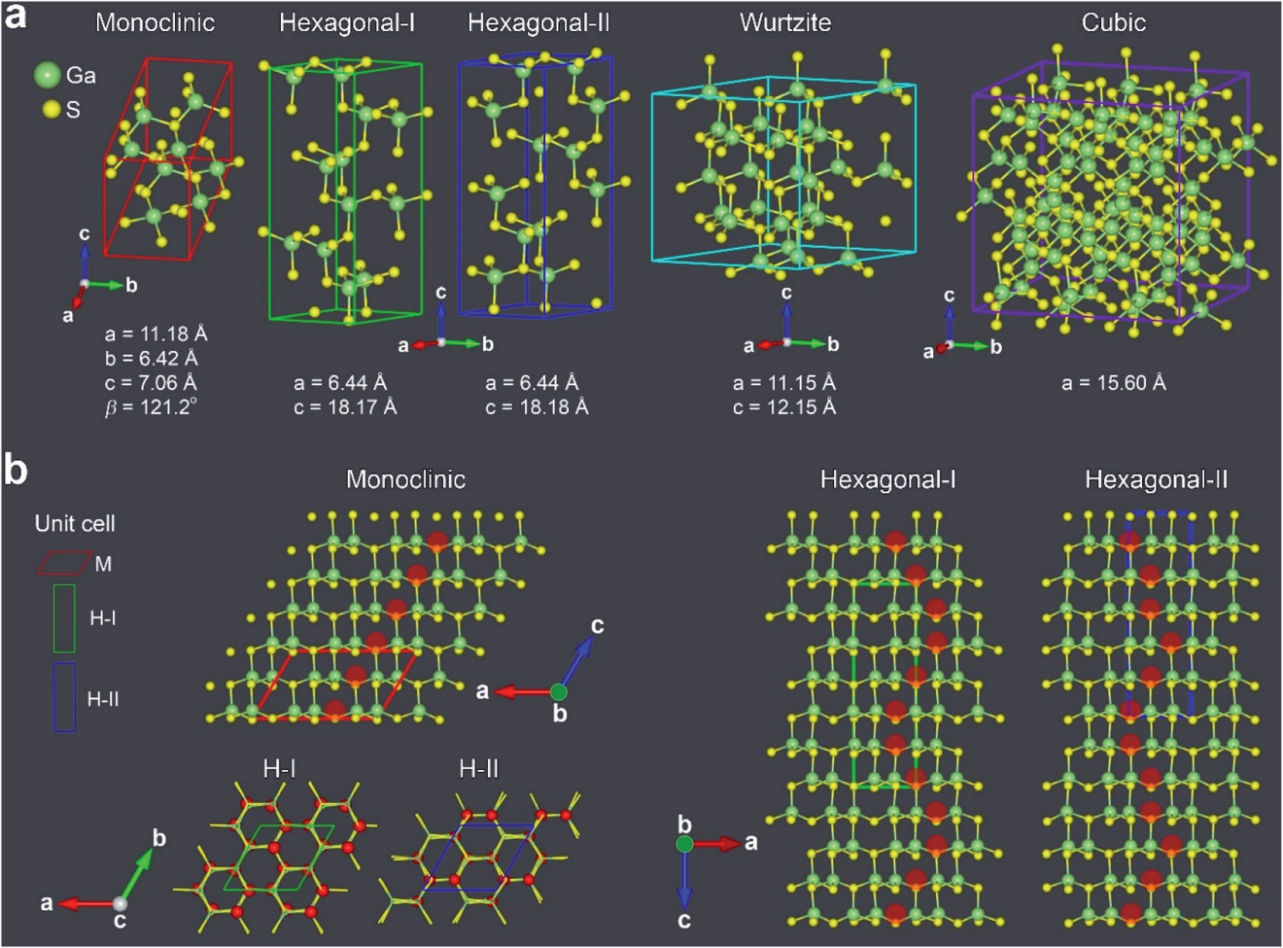
(a) Structures of monoclinic (M), hexagonal (H-I and H-II), wurtzite (W), and zinc blende cubic (C) phases, together with their lattice constants. (b) Atomic arrangement of M, H-I, and H-II structures projected at the [010]_M_ (=[1̄21̄0]_H_) zone axis, revealing the ordered Ga vacancy sites (marked by the red-shaded discs). The H-I and H-II structures are projected at the [0001]_H_ zone axis. Only H-I shows a screw sixfold screw type Ga vacancy (red balls) ordering. The green balls represent Ga atoms while the yellow balls represent S atoms. The unit cell of each phase is outlined in color.

In order to support the phase evolution, we performed density functional theory (DFT) calculations using a supercell geometry with 20–180 atoms. Fig. 4a shows the optimized crystal structures for the M, H-I, H-II, W, and C phases of Ga_2_S_3_. For the M phase, the unit cell contains 8 Ga and 12 S atoms. The H phase was constructed using one H unit cell having Ga_12_S_18_ stoichiometry. For the W and C phases, the supercell was constructed from (3 × 3 × 2) and (3 × 3 × 3) unit cells with Ga_24_S_36_ and Ga_72_S_108_ stoichiometry, respectively. Since the Ga vacancy sites are unknown, we tried to find an optimized configuration using the supercell. The lattice constants of the supercell were within 0.7% difference of the experimental values.

Fig. 4b shows the atomic arrangement of the M, H-I, and H-II structures projected at the [010]_M_ (=[1̄21̄0]_H_) zone axis. The ordered Ga vacancy sites are marked by the red-shaded discs. In the M structure, the Ga vacancies are formed uniformly along the *c* direction, which perfectly depicts the M phase. The H-I and H-II structures exhibit the ordered Ga vacancy sites at the different positions along the *c* direction, which matches the growth direction of Ga_2_S_3_ NWs. The distinctive zigzag pattern is consistent with the experimental data. Top view of H-I (zone axis = [0001]_H_) reveals the 6 Ga vacancy sites that were generated by a sixfold screw rotation with either right-handed (R) or left-handed (L) direction. In contrast, the Ga vacancies of H-II exhibit no such screw feature. The Ga vacancy sites of W and C phase are shown in Fig. S9.†

The calculated total energy confirms that the M phase is the most stable one. The relative energy per Ga_2_S_3_ unit is 0.58 (H-I), 9.18 (H-II), 26.40 (W), and 389.82 meV (C), respectively, and therefore the stability order should be M > H > W > C. The M, H-I, H-II, and W phases are much more stable than the C phase. Furthermore, we calculated the formation energy (*E*_f_) of each phase using the chemical potential of Ga and S atoms (at 0 K). The M, H-I, H-II, W, and C phases exhibit *E*_f_ = −5.103, −5.101, −5.095, −5.078, and −4.685 eV per Ga_2_S_3_ unit, respectively, following the same order of the relative energy. Overall, the total energy calculation can explain the successive phase evolution from the thermodynamically stable M phase to the metastable phases.

The mBJ band structures predicted bandgaps with an accuracy similar to the hybrid functionals or even to GW methods.^39,40^ Our calculation of mBJ band structure suggests that the direct bandgap at the *Γ* point is 1.92, 2.04, 2.07, and 2.11 eV, respectively, for M, H-I, H-II, and W phases (see Fig. S10†). The similar band gap of four phases (∼2 eV) is in reasonable agreement with our experimental values (3 eV). Since the unit cell of C phase consisted of 180 atoms, the bandgap was estimated using DOS, that is 1.41 eV. The PBE calculation predicted the respective values of 1.69, 1.68, 1.72, 1.81, and 0.30 eV, since it generally underestimates the band gap.

**Fig. 5:**
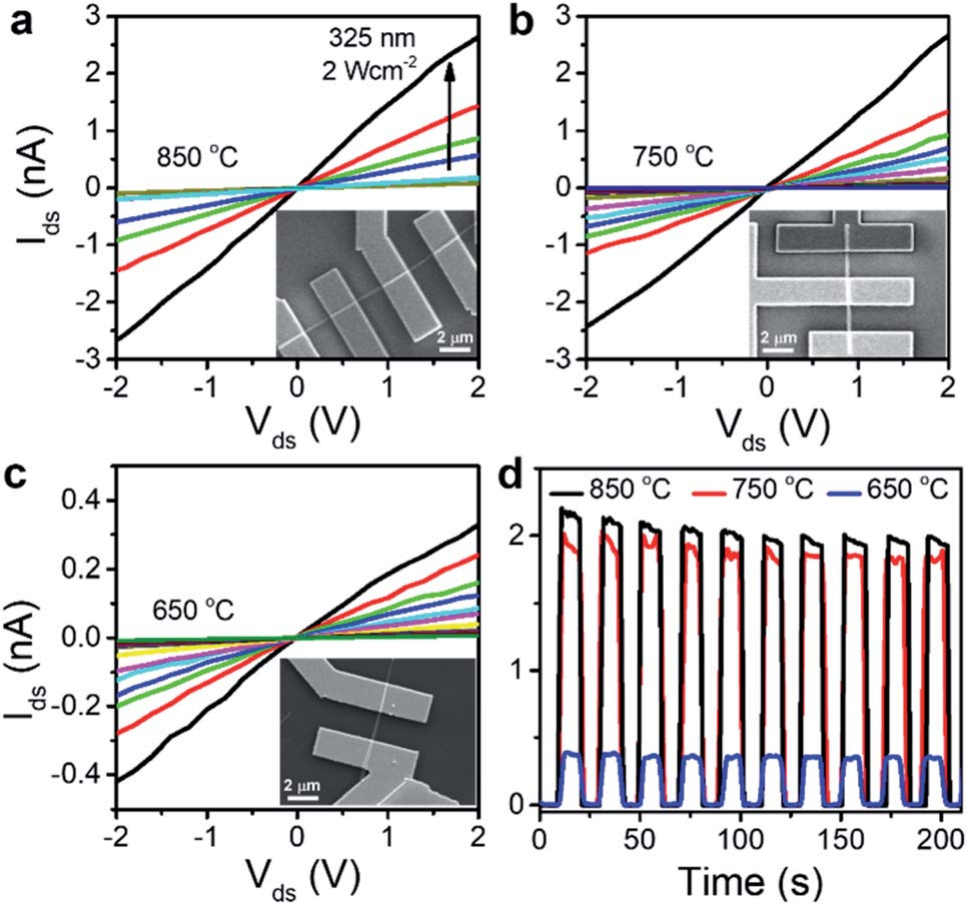
Ga_2_S_3_ NW-based photodetectors. Current–voltage (*I*_ds_–*V*_ds_) characteristics of electrodes fabricated with Ga_2_S_3_ NWs prepared at (a) 850, (b) 750, and (c) 650 °C. Data were measured under 325 nm (He–Cd laser) irradiation at various laser intensities (power density: 0.02–2 W cm^−2^). Insets: SEM images of the Ga_2_S_3_ NW photodetector devices. (d) *I*_ds_–*t* curves under chopped irradiation (10 s intervals) for NWs grown at 850, 750 °C, and 650 °C.

Now, we investigate the dependence of photoelectrical properties on the phases. Fig. 5a–c show the SEM images of photodetector devices fabricated using the NWs, as well as the linear *I*–*V* curves of source-drain (ds) measured under irradiation. The laser power density (*P*) is 0.02–2 W cm^−2^. The dark current (*I*_dark_) was 0.1 pA at *V*_ds_ = 2 V. The NWs grown at 850 and 750 °C exhibited similar current changes upon light irradiation; however, the change was less for the device with NWs grown at 650 °C. Fig. 5d depicts the *I*_ds_–*t* curves for 10 light on–off cycles (10 s each), showing excellent stability and repeatability of the photocurrent. The rise/decay times were shorter than 1 s, and the photocurrent (*I*_p_ = *I*_light_ − *I*_dark_) at 2 V was 2 nA for 850/750 °C and 0.4 nA for 650 °C.

Fig. S11† shows that the photocurrent increases almost linearly with the laser powder density, indicating that the Ga_2_S_3_ NW device possesses a highly efficient photoelectric conversion capability. The linear feature and the fast photocurrent response are probably related to the negligible trapping or scattering of hot carriers, which occurs at the surface defects and the interface between the NW surface and electrodes. The lower photocurrent level in NWs grown at lower temperatures is ascribed to the defect sites that act as trapping sites to decrease the concentration of photogenerated carriers. We suggest that the lattice mismatching C phase against the H/W phase produces the defect sites.

The photosensitivity (*I*_p_/*I*_dark_) was 2 × 10^4^ for the NWs grown at 850 °C. The spectral responsivity (*R*) is defined as the photocurrent generated (*I*_p_) when light of unit intensity shines on the effective area of NW: *R* = *I*_p_/*PA*, where *P* is the incident light intensity (2 W cm^−2^) and *A* is the effective area of the NW (200 nm in diameter and 2 μm in length). We obtained *R* = 1.8 × 10^4^ A W^−1^. Another figure of merit of a photodetector is its specific detectivity (D*), which is defined as D* = *R* (*A*/2*eI*_dark_)^1/2^ when the noise from the dark current is small. We obtained D* = 3.5 × 10^13^ Jones (*i.e.*, cm Hz^1/2^ W^−1^). Table S1† compares the parameters of our devices and previously reported photodetectors based on Ga_2_S_3_, demonstrating the excellent performance of our devices.

## Conclusions

We synthesized high-crystalline Ga_2_S_3_ NWs on Au-deposited Si substrates by a CVD method utilizing the thermal evaporation of Ga_2_S_3_ powders. Decreasing the growth temperature from 850 °C to 600 °C induced sequential phase transitions from the M phase to the H, W, and C phases (M → H → W → C). The M-phase NWs grown at the highest temperature are single crystalline with the growth direction of [001]_M_. For the first time, we found two different H phases (H-I and H-II) with 1.8 nm periodic superlattice structures. The H-I and H-II phase exhibit the ordered Ga vacancy sites at different position along the growth direction. The H-I phase consists of three variants of the M phase, that is, the [010]_M_, [11̄0]_M_, and 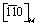. In H-II, the three variants are [010]_M_, 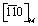, and 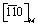 projections. As the temperature decreases, conversion to the W and C phase is accompanied by evolution of the growth direction to [01̄10]_W_ (=[1̄10]_C_) and [12̄10]_W_. The polymorphism of Ga_2_S_3_ produced distinctive core–shell structures that illustrate the phase evolution occurred by sharing the same crystallographic axis within the NWs. Using the DFT, extensive total energy calculations were carried out on four types of crystal structures, showing that the stability follows the order of M > H > W > C, which support the experimental phase transition. The H-I and H-II phases exhibit consistently the ordered Ga vacancy sites with a similar stability. The photodetector devices showed that the photoresponsivity can reach 1.8 × 10^4^ A W^−1^. These results suggest a new strategy for enhancing the performance of NW-based UV photodetectors.

## Author contributions

K. P.: investigation (lead), synthesis and crystal analysis, D. K.: device fabrication, T. T. D.: calculation, M. B: calculation, G. M. Z.: calculation (supporting), J. S.: synthesis, I. S. K.: characterization, I. H. K.: characterization, M. K. J.: supervision (supporting), J. P.: project lead, writing, H. S. K.: calculation lead.

## Conflicts of interest

There are no conflicts to declare.

## Supplementary Material

NA-004-D2NA00265E-s001
