# Supplementary material for: Polymorphic Ga2S3 nanowires: phase-controlled growth and crystal structure calculations
Source: Nanoscale Adv. 2022 Jul 1;4(15):3218–25. doi: 10.1039/d2na00265e (PMC9419741; doi:10.1039/d2na00265e)
Supplement: NA-004-D2NA00265E-s001 [file NA-004-D2NA00265E-s001.pdf]

Electronic Supplementary Material (ESI) for Nanoscale Advances.
This journal is © The Royal Society of Chemistry 2022

Electronic Supplementary Information.

Polymorphic Ga_2S_3 Nanowires: Phase-Controlled Growth and Crystal Structure Calculations

Kidong Park,[a] Doyeon Kim,[a] Tekalign Terfa Debela,[b] Mourad Boujnah,[b] Getasew Mulualem Zewdie,[b] Jaemin Seo,[a] Ik Seon Kwon,[a] In Hye Kwak,[a] Minkyung Jung,[c] Jeunghee Park,*[a] and Hong Seok Kang*[d]

[a] Department of Advanced Materials Chemistry, Korea University, Sejong 339-700, Republic of Korea; [b] Institute for Application of Advanced Materials, Jeonju University, Chonbuk 55069, Republic of Korea; [c] DGIST Research Institute, DGIST, Daegu 42988, Republic of Korea; [d] Department of Nano and Advanced Materials, Jeonju University, Chonju, Chonbuk 55069, Republic of Korea

K.P., D.K., and T.T.D. contribute equally as first author.

Content

Experimental details

Table S1. Comparison of our photodetector performance with previous reported works.

Figure S1. Schematic diagram of unit cells and the correlation of crystallographic axes.

Figure S2. SEM micrographs of Ga_2S_3 NWs.

Figure S3. SEM/HRTEM images of Ga_2S_3 NWs grown at 850 and 650 °C, and growth mechanism.

Figure S4. XPS data of Ga_2S_3 NWs.

Figure S5. Raman spectrum of Ga_2S_3 NWs.

Figure S6. UV-visible spectrum and PL spectrum of Ga_2S_3 NWs.

Figure S7. Atomic arrangement of M phase crystal upon the rotation around the $[001]_M$ axis.

Figure S8. Schematic diagram of growth direction evolution.

Figure S9. Crystal structures of W and C phases.

Figure S10. The mBJ band structures and DOS.

Figure S11. Photocurrent *vs.* laser intensity.

References

Experimental Details

(1) Synthesis

Ga_2S_3 (99.99 %, Alfa Aesar) powders were placed in ceramic boats, which are loaded inside a quartz tube CVD reactor that is heated using an electrical furnace. A silicon (Si) substrate, on which a 5 nm-thick Au film was deposited, was positioned at a distance of 18 cm away from the powder source. The reactor was evacuated using a mechanical pump. Then argon gas is continuously supplied at a rate of 500 sccm during growth while the pressure maintains below 20 Torr. The temperature of the powder sources is set to 900-950 °C. The substrate is maintained at 600-850 °C to synthesize the nanowires. The temperature of the source and substrate as well as

Sample No.	Temperature of source (°C)	Temperature of substrates (°C)	Reaction time (min)
1	950	850	10-40
2	950	800	10-40
3	950	750	30-60
4	950	700	30-60
5	900	650	60-120
6	900	600	60-120

the reaction time for each sample was summarized as follows.

(2) Characterization

The structure and composition of the products were analyzed by scanning electron microscopy (SEM, Hitachi S-4700), field-emission transmission electron microscopy (TEM, FEI TECNAI G^2

200 kV), high-voltage TEM (HVEM, Jeol JEM ARM 1300S, 1.25 MV), and energy-dispersive X-ray fluorescence spectroscopy (EDX). Fast Fourier-transform (FFT) images were generated by the inversion of the TEM images using Digital Micrograph GMS1.4 software (Gatan Inc.). A tilt holder (Dual Orientation Tomography Holder 927, Gatan Co.) was used for the TEM measurements. High-resolution XRD patterns were obtained using the 9B beam line of the Pohang Light Source (PLS) with monochromatic radiation. XRD pattern measurements were also carried out in a Rigaku D/MAX-2500 V/PC using Cu K_α radiation (λ = 1.54056 Å). X-ray photoelectron spectroscopy (XPS) data were collected using the 10A2 beam line of the PLS with photon energy of 380 and 630 eV. XPS measurements were also performed using a laboratory-based spectrometer (Thermo Scientific Theta Probe) using a photon energy of 1486.6 eV (Al Kα). A UV-Vis-NIR spectrometer (Agilent Cary 6000) was used to measure the UV-visible absorption of samples. Raman spectra were collected on a home-made Raman system with 514.5 nm (2.41 eV) line from an Ar ion laser (Melles Griot 43 series ion laser, 543 AP-A01). The laser power on the sample was kept below 1 mW to avoid destroying or heating the sample. A 100× objective and charge-coupled device (CCD) detector (Princeton Instruments Acton sp2500, spectral resolution was about 1 cm^{-1}) were used to collect Raman signals.

(3) Fabrication of photodetector and field-effect transistor (FET) devices

A highly doped p^{++} Si (B-doped with a resistivity of < 0.005 Ω·cm) substrate with a 300-nm-thick thermally grown silicon oxide layer on top was prepared. Photolithography was used to deposit Ni (20 nm)/Au (130 nm) electrodes on the substrate by sputtering using a patterned mask. The NWs were dispersed by applying a drop of its isopropyl alcohol (IPA) solution onto a patterned Si substrate. The samples were then coated with a layer of poly(methyl methacrylate) (PMMA, A4, Microchem. Co.), and the electrode patterns were created in an electron-beam lithography process.

After developing, the samples were loaded in an electron beam evaporator, and 20 nm of Ni was deposited, followed by 130 nm of Au. After the lift-off process, the samples were usually annealed to remove the fabrication residues. The electrical transport properties were measured in a commercial probe station (WWIT Co. VPX-10) with parametric test equipment (Agilent E5270A). The p^{++} Si substrate was used for a back gate of FET devices, and the gate effect was measured at ambient conditions (300 K), and the potential was applied using the Au probe tip (diameter = 5 μm). The photocurrent measurement was performed using 325 nm (3.81 eV) from a He-Cd laser (KIMMON IK Series, IK3401R-F).

Table S1. Comparison of photosensitivity, responsivity, and detectivity of Ga_2S_3 NW photodetector with previous reported work on Ga_2S_3 and Ga_2Se_3.

Materials	λ (nm)	Applied Voltage (V)	I_p [b]	P [c]	R ($A\ W^{-1}$) [d]	R* (Jones) [e]	Ref.
α′-Ga_2S_3	405	V_{ds} = 1000	25 nA	0.98 [a]			S1
α′-Ga_2S_3/GaAs	405	V_{ds} = 4	2.3 μA	14			S2
α′-Ga_2S_3 nanosheets	455	V_{ds} = -5	40 μA	--	240	10^{10}	S3
γ-Ga_2S_3 nanoflakes	350	V_{ds} = 1	1 μA	851	61.3	$^{-}1.52 \times 10^{10}$	S4
α-Ga_2S_3 nanosheets	355	V_{ds} = 2	46.2 nA	76	9.2	1.4×10^{12}	S5
β-Ga_2Se_3 NWs	365	V_{ds} = 30	0.05 nA	250	707	2.9×10^{12}	S6
Cubic β-Ga_2Se_3 plate	642	V_{ds} = 30	1 nA	1000 [a]	3.24	2.5×10^{10}	S7
α′-Ga_2S_3 NWs	325	V_{ds} = 2	2 nA	2×10^4	1.8×10^4	3.5×10^{13}	Present work

[a] We estimated the value using the data in the text.

[b] Photocurrent (I_p) is I_{light}-I_{dark}, where I_{light} and I_{dark} is the current under light and dark condition,

[c] Photosensitivity (P) defined as I_p/I_{dark}.

[d] Spectral responsivity (R), defined as the photocurrent generated when light of unit intensity shines on the effective area of NW or 2D nanosheet, can be expressed as $R = \Delta I/PA$, where P is the incident light intensity and A is the effective area.

[e] Specific detectivity (D*) in a unit of Jones (i.e., $cm \cdot Hz^{1/2} W^{-1}$). When the noise from dark current is small, it can be defined as $D^* = R\ (A/2eI_{dark})^{1/2}$.

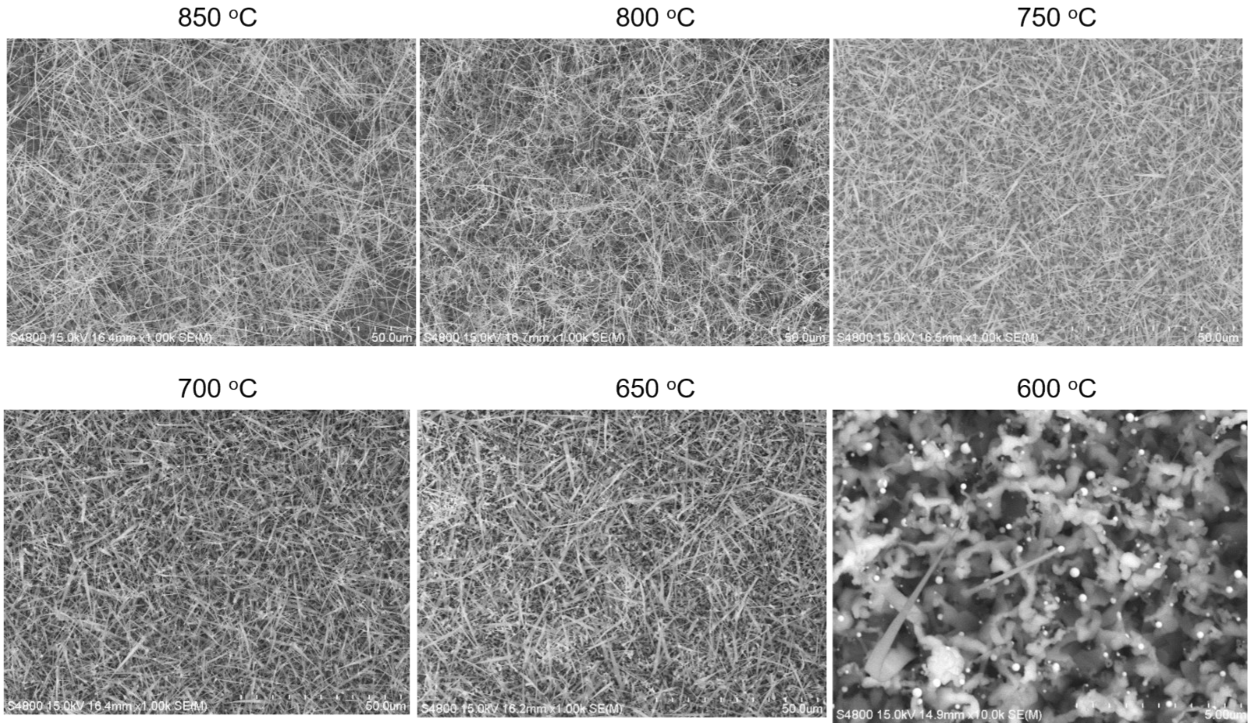

Figure S1. SEM images of Ga_2S_3 NWs grown at various temperatures.

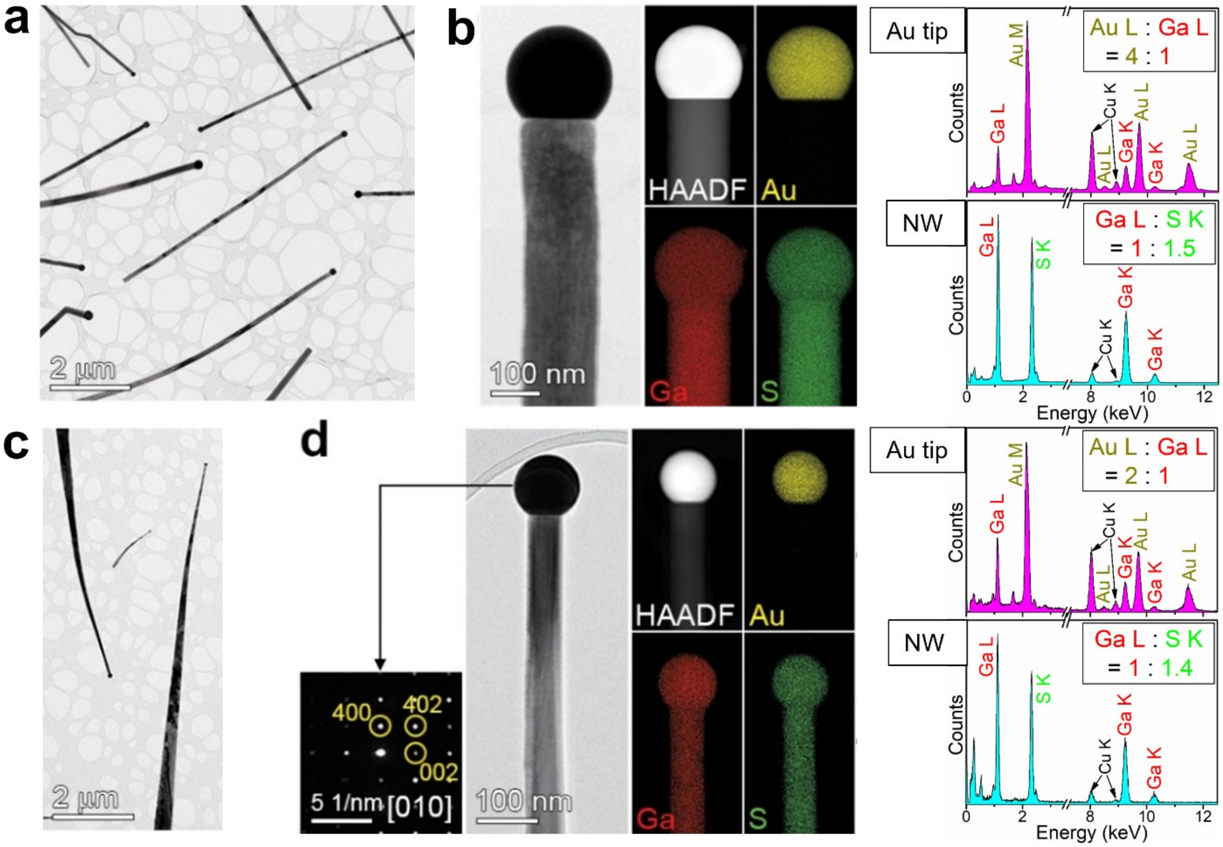

Figure S2. HRTEM images of Ga_2S_3 NWs grown on a Si substrate at (a) 850 °C and (c) 650 °C, showing high-density Ga_2S_3 NWs with Au at the tip. HAADF STEM images, EDX elemental mapping of Ga L shell, S K shell, and Au L shell peaks, and the corresponding EDX spectra for the NW body and Au catalyst tip grown at (b) 850 °C and (d) 650 °C. The SAED pattern (zone axis = [010]) in (d) corresponds to the Au tip consisting of orthorhombic phase Au_2Ga.

At 850 °C, the Ga_2S_3 NWs had a straight and smooth surface. The diameter of the NWs (average value: 150 nm) was uniform along a few tens of micrometers in length (Figure S2a). Au nanoparticles (NPs) with a size of 230 nm were attached to the ends of the NWs (Figure S2b). The high-angle annular dark-field scanning TEM (HAADF-STEM) image and energy-dispersive X-ray spectroscopy (EDX) elemental mapping of the Ga L shell, S K shell, and Au L shell revealed a homogeneous distribution of Ga and S in the whole NW. The S K shell overlapped with the Au M shell, and so its mapping covered the Au NPs. The atomic ratio between Ga and S in the NW was 1.5. At the tip, the atomic ratio of Au:Ga is approximately 4:1 (25% Ga), which is close to the

Au_7Ga_2 alloy phase.

At 650 °C, the width of the NW was gradually reduced by more than half when approaching the Au tip (Figure S2c). The size of the Au tip was not larger than 100 nm. Compared to the case at 850 °C, this reduced Au tip size is due to the lower temperature that would retard the agglomeration of Au. The HAADF-STEM image and EDX elemental mapping revealed that the spherical Au-Ga alloy NPs with a size of 100 nm were attached to the NWs with a diameter of 50 nm (Figure S2d). The selected-area electron diffraction (SAED) pattern of the tip indicated orthorhombic phase Au_2Ga (JCPDS No. 29-0619; $Cmc2_1$, a = 18.02 Å, b = 3.199 Å, and c = 6.999 Å). The diffraction spots were assigned to the zone axis of [010]. The atomic ratio of Au/Ga was 2:1 (33% Ga), corresponding to the composition of Au_2Ga. The Ga:S ratio of the NWs was approximately 1.5.

Now, we discuss the growth mechanism of Ga_2S_3 NWs. The round shape of the catalyst NPs indicates that the NW growth follows the typical vapor-liquid-solid (VLS) mechanism. As the precursors supply Ga and S vapors, Ga dissolves into the Au NPs by forming Au-Ga eutectics, whereas the concentration of S in the NPs is negligibly small owing to its low solubility. Saturated Ga precipitates with S vapors in the triple-phase region (*i.e.*, the interface among the NW solid, the Au alloy liquid droplet, and the gas phase reactants) to produce the NWs. According to the phase diagram, more Ga can melt in Au at a lower temperature because of the eutectic points of Au_7Ga_2 (410 °C) and Au_2Ga (350 °C). The smaller size of the Au droplet facilitates efficient dissolution and precipitation of Ga. Then, this high Ga concentrations would allow the growth of metastable phase NWs, corresponding to a case of kinetically controlled growth. The conversion into metastable phases ($M \rightarrow H \rightarrow W \rightarrow C$) is driven by the richer Ga at a lower temperature. The tapered belt-like morphology (shown in Figure S3d) indicates kinetically dominated precipitation through the sidewalls. The termination of growth (followed by a slower growth rate) reduces the NW diameter.

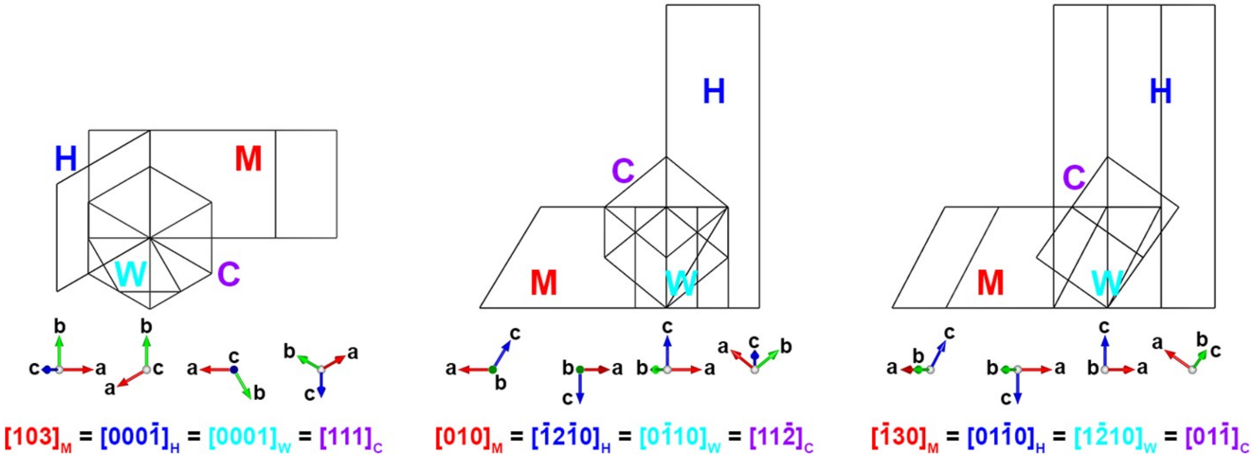

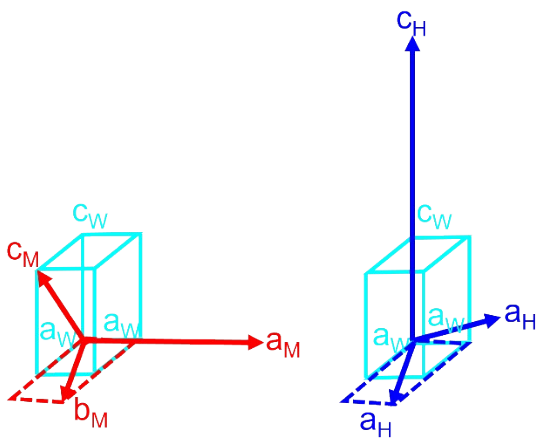

$a_M = 3a_W$, $b_M = \sqrt{3}a_W$, and $c_M = \sqrt{a_W^2 + c_W^2}$

$a_H = \sqrt{3}a_W$ and $c_H = 3c_W$

Figure S3. Schematic diagram showing the correlation between the unit cells and crystallographic axes, for monoclinic (M), hexagonal (H), wurtzite (W), and zinc blende cubic (C) phases.

₩

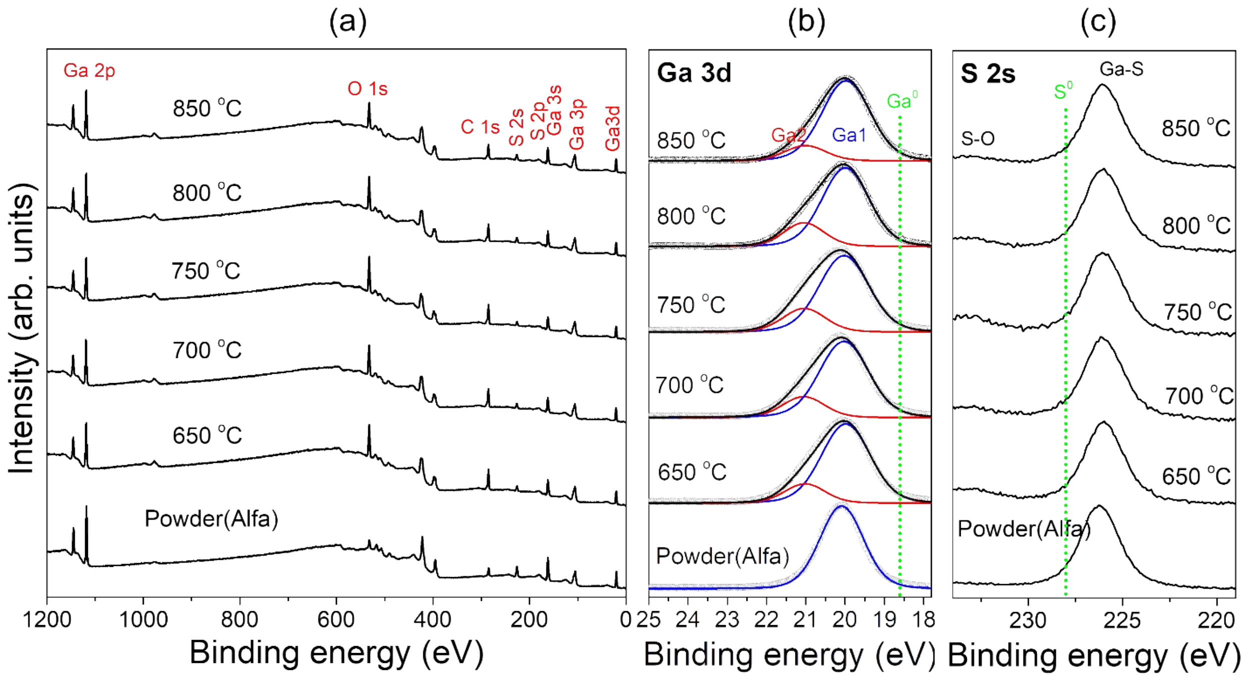

Figure S4. (a) Survey scanned XPS spectrum for Ga_2S_3 NW samples (650-850 °C) and powders (monoclinic phase bulk crystal purchased from Alfa). Fine scanned (b) Ga 3*d* and (b) S 2s peaks. The data (open circles) are fitted by a Voigt function, and the sum of the resolved bands is represented by a black line. The positions of the peaks corresponding to neutral species ($3d_{5/2}$ of Ga^0 and 2s of S^0) are marked by dotted lines to highlight the corresponding shift.

The survey scan shows Ga and S peaks. Fine-scanned Ga 3d peaks whose $3d_{3/2}$ and $3d_{5/2}$ (separated by 0.44 eV) are unresolved. The Ga_2S_3 powders show the peak at 20 eV, corresponding to the Ga-S bonding, which are blue shifted from the Ga metal (Ga^0) at 18.6 eV. The peaks of NW samples are asymmetric, and thus resolved into two components (Ga1 and Ga2), using a Voigt function: Ga1 (blue) at 20 eV and Ga2 (red) at 21 eV. The Ga1 and Ga2 were assigned to the Ga-S and Ga-O, respectively. The fraction of Ga-O band was determined as 10-15%, indicating that the presence of surface oxidation. The XPS S 2s peak at 226 eV, redshifted from that of neutral S (S^0 at 228 eV), is attributed to the Ga-S bonding structures. The NW samples shows a weak peak at 233 eV (gray shade region), which can be assigned to the S-O bonds, while the Ga_2S_3 powders exhibit no peak. We conclude that Ga_2S_3 NWs have some surface defects originated from the oxide layers.

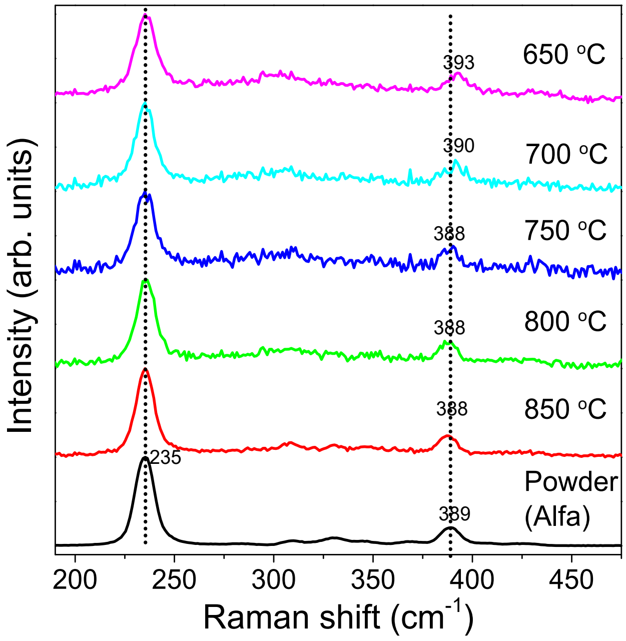

Figure S5. Raman spectrum of Ga_2S_3 NWs synthesized at 650-850 °C and bulk powders. Raman signals were obtained using an Ar ion laser with a wavelength of 514.5 nm.

Micro-Raman spectra were measured for the individual NWs using a 514.5-nm excitation source. The monoclinic phase bulk crystal (powder purchased from Alfa) and the NW samples shows two broad peaks at 235 and 389 cm^{-1}. They originated from the breathing (A_1) and the symmetric stretching (F_2) vibrational modes, respectively, in the GaS_4 molecular unit of wurtzite phase structures which exhibit tetrahedral bonding and one-third Ga vacant sites (on the average) around the chalcogen atoms.[S1,S8,S9] In the breathing mode, the four neighbors of vacancy move together along the anion-vacancy direction.[S10] The maintenance of two Raman peaks for all samples indicates the same wurtzite unit. The NWs grown at 750, 800 and 850 °C show the A_1 and F_2 mode peaks at the same position; 235 and 388 cm^{-1}, respectively, which are close to those of the bulk crystal powders. The monoclinic and hexagonal phase structures are superstructures of the wurtzite phase, as shown Figure S3. Therefore, Raman peaks of three phase are the same. The F_2 vibration mode (390 and 393 cm^{-1}) shows a blueshift at the lower growth temperature (700 and 650 °C, respectively) is ascribed to the shorter Ga-Ga bond distance since the lattice constant of C phase is smaller than that of W phase by 0.5%, based on the W-C phase relationship.

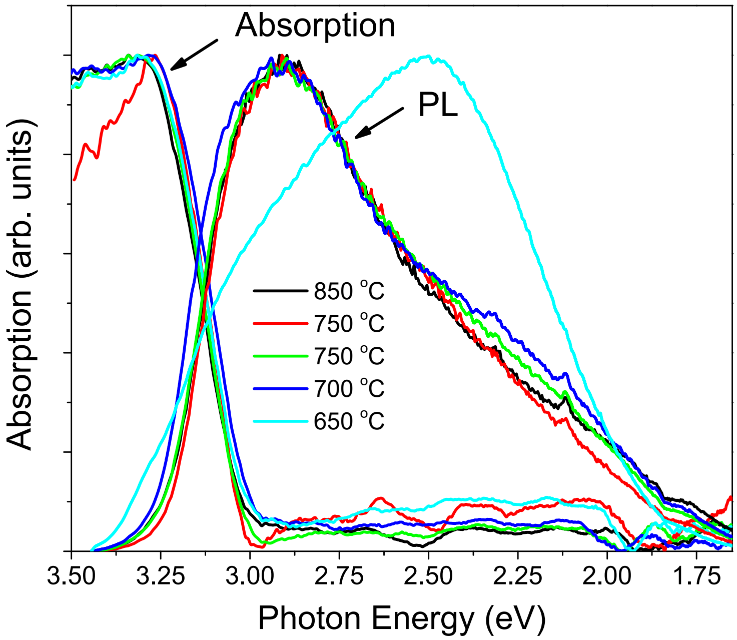

Figure S6. UV-visible diffuse reflectance spectrum (in absorption) of Ga_2S_3 NW samples (grown at 650-850 °C). Photoluminescence (PL) spectrum (normalized) measured at room temperature using the excitation energy of 3.81 eV (325 nm He-Cd laser).

For all NW samples synthesized at different temperatures, the onset of UV-visible spectrum appears at 3.0 eV, suggesting that the optical band gap (E_g) value is 3.0 eV. As the temperature decreases, the broad band (center = 2.4 eV) increases in intensity. The micro-PL spectrum was measured by delivering continuous-wave excitation from a 325 nm He-Cd laser to individual NW. It shows the band-edge peak at 2.9 eV and the broad defect emission centered at 2.3 eV (in the range of 1.6-2.6 eV). As the growth temperature decreased, the PL intensity significantly decreases by a factor of 10. The broad absorption and PL band which would be originated from the Ga and/or S defect sites.[S1,S3,S11] The enhanced defect peaks at 650 °C is due to the incorporation of the C phase that has the smaller lattice constant of C phase is than that of H/W phase by 0.5%.

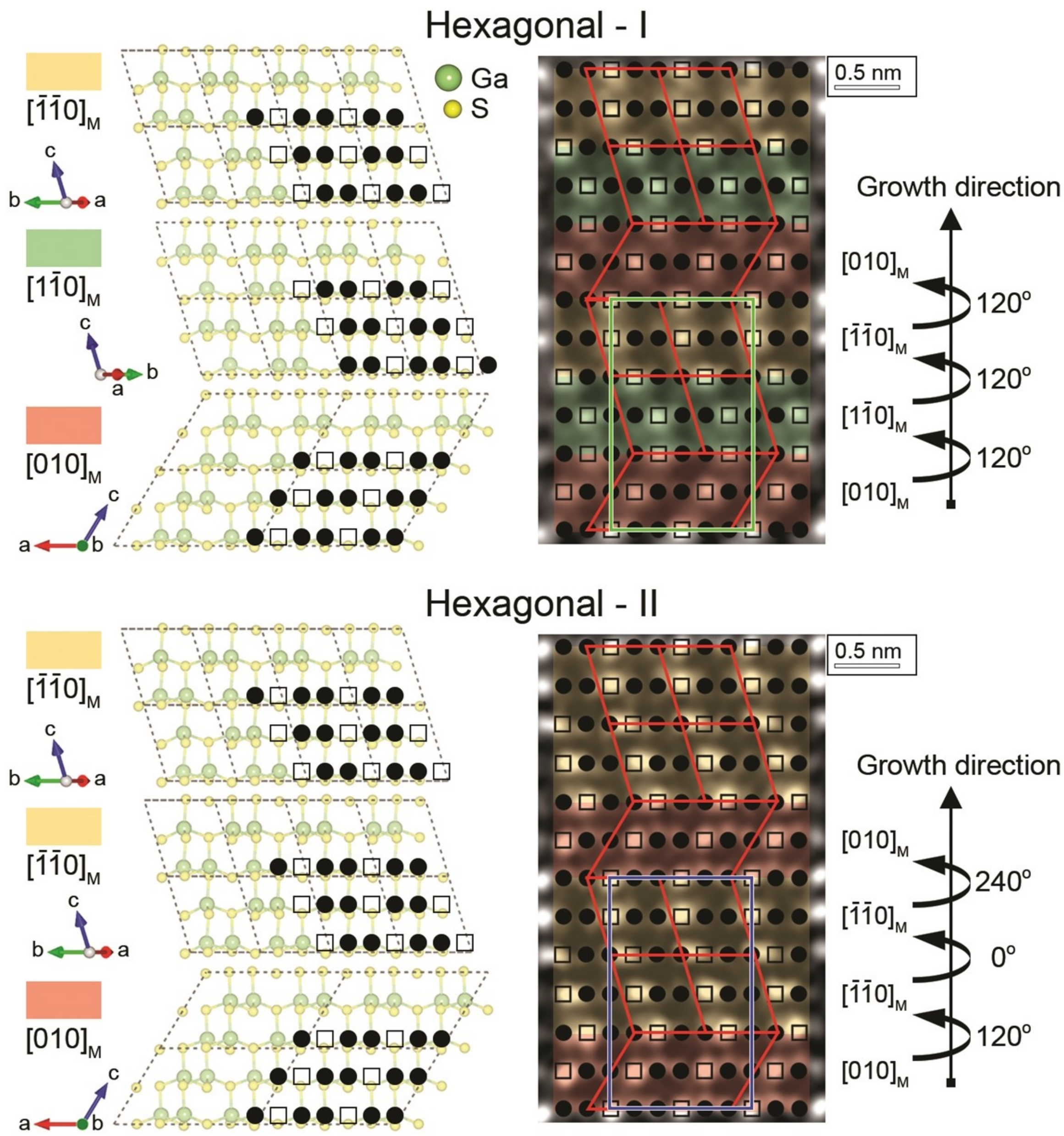

Figure S7. Atomic arrangements of hexagonal-I (H-I) structures consisted of $[010]_M$ (red), $[10]_M$ (green), and $[0]_M$ (yellow) projections by virtue of rotating 120° around the $[001]_M$ rotation axis. Hexagonal-II (H-II) structures consisted of $[010]_M$ (red), $[0]_M$ (yellow), and $[0]_M$ (yellow) variants projected by 120° and 0° rotation. Filled circle and hollow square represent sites occupied by Ga and vacancies, respectively.

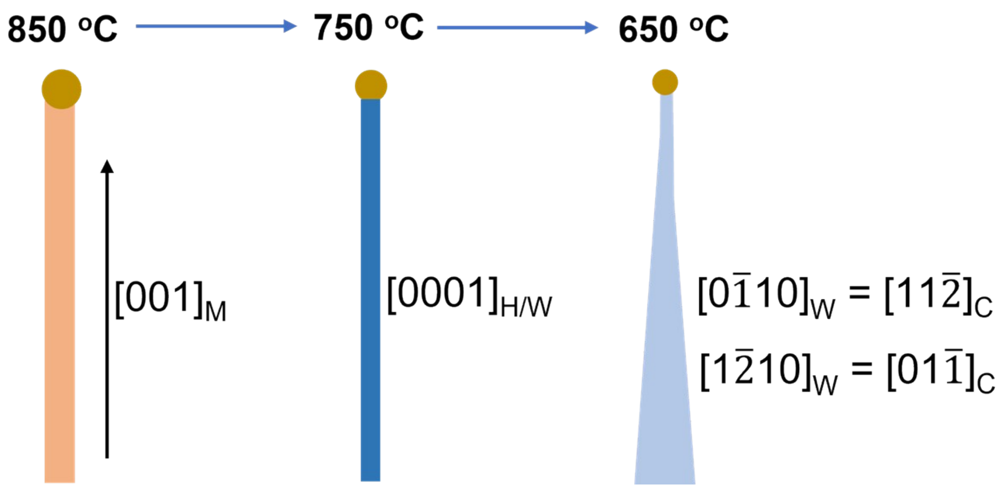

Figure S8. Schematic diagram for the morphology and growth direction of Ga_2S_3 NWs grown at various growth temperatures.

As the growth temperature decreases, the conversion from the M phase into metastable phases (M → H → W→ C) occurs and the NW morphology changed from a cylindrical (750 and 850 °C) to a tapered belt shape (650 °C). The growth direction of the cylindrical NWs is $[0001]_{H/W}$. The tapered belt-like NWs have the growth direction is either $[010]_W$ or $[110]_W$. The crystallographic axes of the four phases link as follows: $[103]_M$ (normal to the shared $(001)_M$ plane) = $[000]_H$ = $[0001]_W$ = $[111]_C$, $[010]_M$ = $[20]_H$ = $[010]_W$ = $[11]_C$, and $[30]_M$ = $[010]_H$ = $[110]_W$ = $[01]_C$.

Here, we suggest a growth model to explain the temperature-dependent growth direction. The crystallographic correlation between the four phases can be transformed to that of the C phase. The growth direction is $[111]_C$ at 750 and 850 °C, and it is $[11]_C$ (main) and $[01]_C$ at 650 °C. In the C phase, the surface energies of the crystallographic planes can be arranged in the following order: $\{111\}_C < \{100\}_C < \{110\}_C$.[S12,S13] In the $[111]_C$ growth direction (at 750 and 850 °C), precipitation occurs mainly at the flat NW-Au interfacial facet ($\{111\}_C$), which requires the lowest Gibbs free energy. The $\{110\}_C$ facets on the side walls provide less favorable precipitation sites. In contrast, the $[01]_C$ growth direction benefits from the side walls with the $\{111\}_C$ facets at the lowest surface

energy. The higher concentration of Ga in the catalyst facilitates precipitation through the side walls, which drives kinetically controlled growth. In the $[11]_C$ growth direction, the $\{111\}_C$ and $\{110\}_C$ facets on the side walls provide a transitional direction between the $[111]_C$ and $[01]_C$ growth directions. If the C phase is more dominant, then the $[01]_C$ growth direction is preferred. Unfortunately, the yield at 600 °C, where C is the dominant phase, was too low for sample analysis. This speculative growth model requires further studies to elucidate the growth mechanism of Ga_2S_3 NWs.

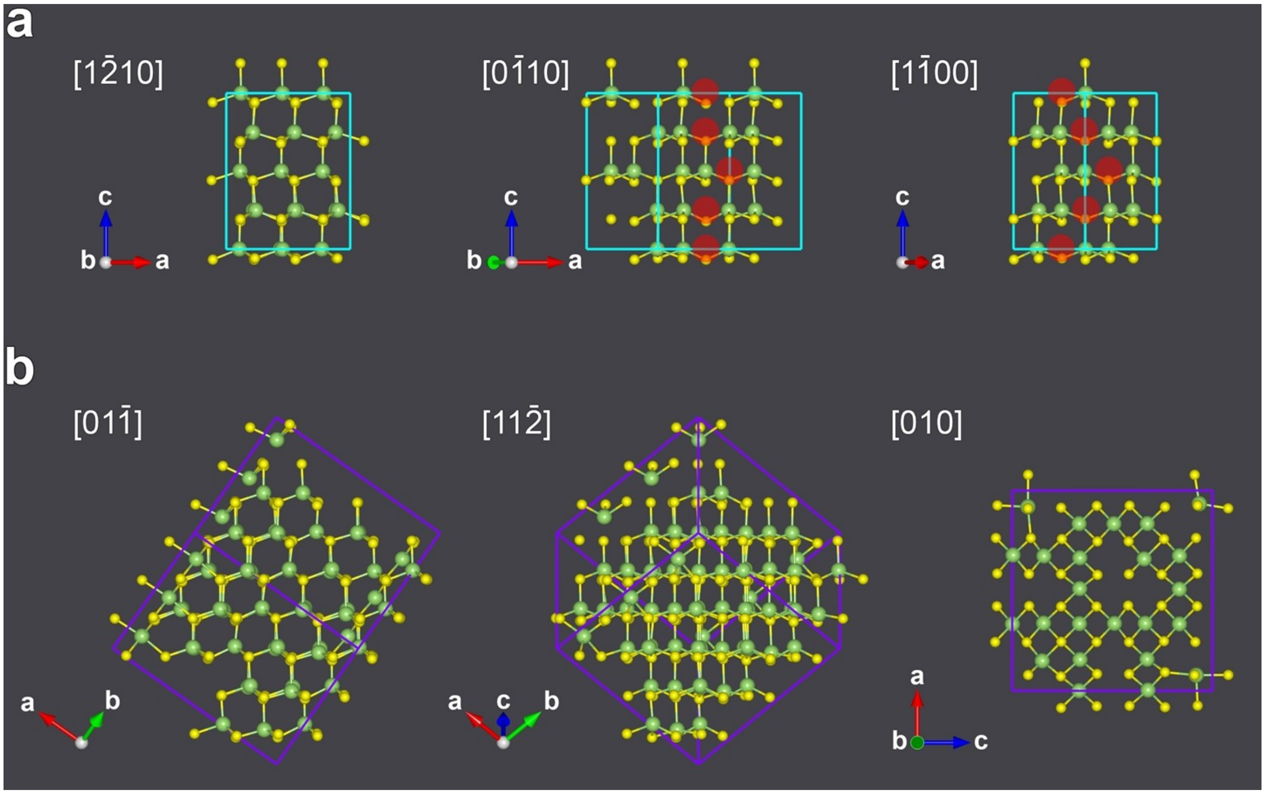

Figure S9. Crystal structure of (a) W phase (3×3×2) unit cells (zone axis = [110], [010] and [100]) and (b) C phase (3×3×3) unit cells (zone axis = [01], [11] and [010]). The Ga vacancies are marked by the red-shaded discs.

For the W phase, the supercell was constructed from (3×3×2) unit cells, where each unit cell has Ga_2S_2 stoichiometry. Next, 12 Ga vacancies were generated in a systematic way as shown in (a). This configuration was vastly more stable than the configurations with random Ga vacancies, *i.e.*, by at least 0.24 eV per Ga_2S_3 unit. For the C phase, the supercell was constructed based on (3×3×3) unit cells, where the underlying zinc blende unit cell corresponded to Ga_4S_4 stoichiometry. Next, 36 Ga vacancies were generated systematically so that they complied with all the symmetry elements of the C phase supercell. This configuration is significantly more stable than random vacancy configurations, *i.e.*, by at least 0.16 eV per Ga_2S_3 unit.

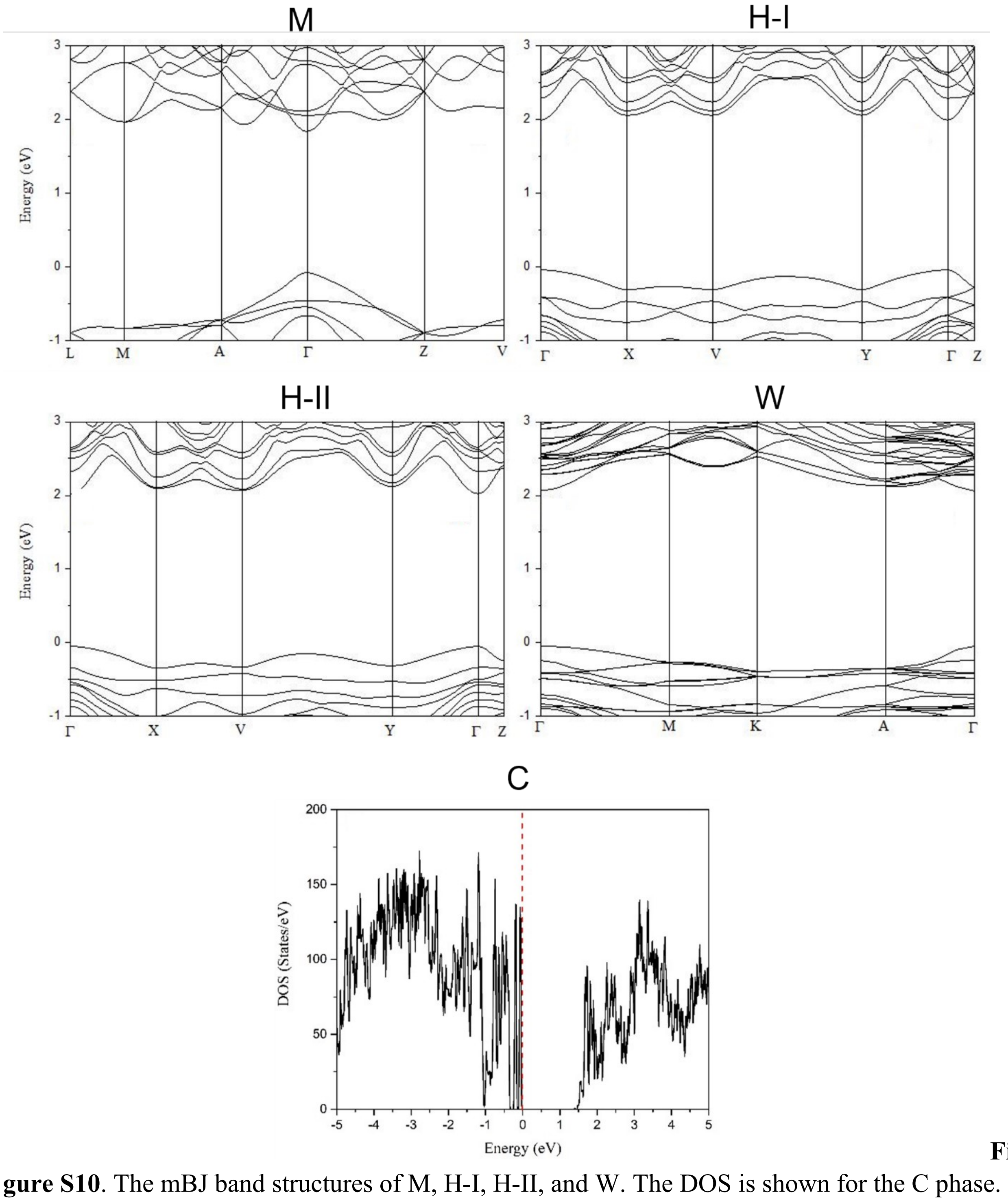

Figure S10. The mBJ band structures of M, H-I, H-II, and W. The DOS is shown for the C phase.

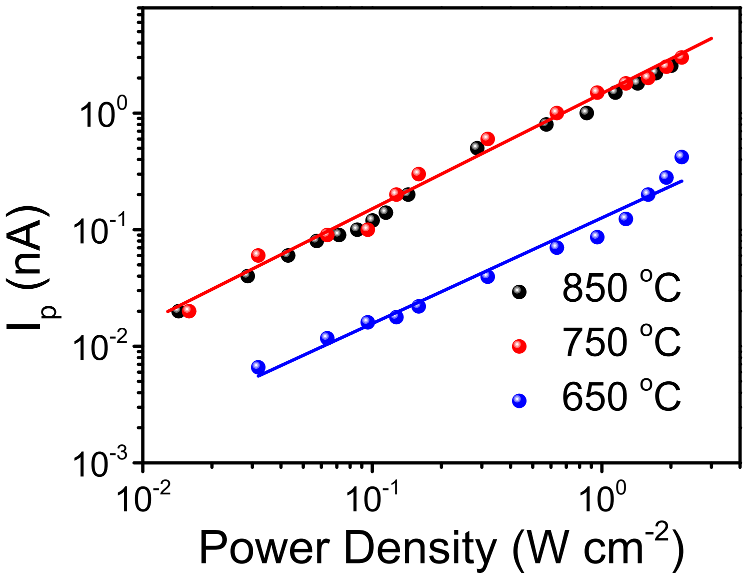

Figure S11. Photocurrent (I_p) *vs.* laser power density (at 325 nm) at a bias voltage of 2 V.

A log-log plot of the photocurrent (I_p) was defined as the current increase upon light irradiation as a function of the laser intensity. The relationship was fitted as $I_p \propto P^\alpha$, where P is the laser power density (W cm^{-2}). At 850 and 750 °C, α = 0.98 in the range of P = 0.02-2 Wcm^{-2}. At 650 °C, α = 0.91. An approximate linear behavior indicates that the Ga_2S_3 NW device possesses a highly efficient photoelectric conversion capability. The unity exponent in the I_p–P relationship and the fast photocurrent response (Figure 5d in maunscript) are probably related to the negligible trapping or scattering of hot carriers, which occurs at the surface defects and the interface between the NW surface and electrodes. The slight decrease of α and the lower photocurrent level in NWs grown at lower temperatures is ascribed to the defect sites that act as trapping sites to decrease the concentration of photogenerated carriers. The lattice mismatching C phase against the H/W phase would produce the defect sites.

References

S1. C. -H. Ho and H. -H. Chen, *Sci. Rep.*, 2014, **4**, 6143.

S2. H. F. Liu, K. K. A. Antwi, N. L. Yakovlev, H. R. Tan, L. T. Ong, S. J. Chua and D. Z. Chi, *ACS Appl. Mater. Interfaces*, 2014, **6**, 3501.

S3. M. M. Y. A. Alsaif, N. Pillai, S. Kuriakose, S. Walia, A. Jannat, K. Xu, T. Alkathiri, M. Mohiuddin, T. Daeneke, K. Kalantar-Zadeh, J. Z. Ou and A. Zavabeti, *ACS Appl. Nano Mater.*, 2019, **2**, 4665.

S4. N. Zhou, L. Gan, R. Yang, F. Wang, L. Li, Y. Chen, D. Li and T. Zhai, *ACS Nano*, 2019, **13**, 6297.

S5. Y. Zheng, X. Tang, W. Wang, L. Jin and G. Li, *Adv. Funct. Mater.*, 2020, **31**, 2008307.

S6. K. Park, J. Lee, D. Kim, J. Seo, J. A. Lee, I. H. Kwak, I. S. Kwon, J. P. Ahn and J. Park, *J. Phys. Chem. C*, 2019, **123**, 3908.

S7. C. H. Ho, *ACS Omega*, 2020, **5**, 18527.

S8. G. Lucazeau and J. Leory, *Spectrochimica Acta*, 1978, **34A**, 29.

S9. C. Julien, S. Barnie, I. Ivanov, M. Guittard, M. P. Pardo and A. Chilouet, *Mater. Sci. Eng. B*, 1999, **57**, 102.

S10. E. Finkman, J. Tauc, R. Kershaw and A. Wold, *Phys. Rev. B*, 1975, **11**, 3785.

S11. T. Aono and K. Kase, *Solid State Commun.*, 1992, **81**, 303.

S12. Y. Wu, Y. Cui, L. Huynh, C. J. Barrelet, D. C. Bell and C. M. Lieber, *Nano Lett.*, 2004, **4**, 433.

S13. V. Schmidt, S. Senz and U. Gösele, *Nano Lett.*, 2005, **5**, 931.
